# Supplementary material for: What Turns a Product into a Traditional One?
Source: Foods. 2021 Jun 4;10(6):1284. doi: 10.3390/foods10061284 (PMC8226589; doi:10.3390/foods10061284)
Supplement: Supplementary file 1 [file foods-10-01284-s001.zip › foods-1218112-supplementary.pdf]

Supplementary File: Objective knowledge for Mezcal and Tequila

Please, select the option you think is correct for each question.

1. Which of the following elements is not present in Mezcal?
a) Alcohols
b) Esters
c) Mezcalina
d) Terpenes
e) I don't know
2. What is the leading state of Mezcal production in the country?
a) Durango
b) Oaxaca
c) Tabasco
d) Mexico City
e) I don't know
3. It is an element that is usually used in the elaboration of artisanal Mezcal.
a) Diffuser
b) Tahona
c) Extruder
d) Concentrator
e) I don't know
4. Which of the following types of process is not used to make Mezcal?
a) Traditional
b) Artisanal
c) Mechanic
d) Industrial
e) I don't know
5. What element protects mezcal production?
a) Designation of origin
b) Sanity Norm
c) ISO Norm
d) Intellectual Protection Norm
e) I don't know
6. It is a sensory characteristic resulting from the cooking process with firewood in the Mezcal
a) Fatty aroma
b) Smoked aroma
c) Alcohol aroma
d) Perfume aroma
e) I don't know
7. Mezcal is a beverage:
a) Foreigner
b) Distilled
c) Concentrated
d) Moisturizing
e) I don't know
8. One of the differences between mezcal and Tequila is:
a) The raw material
b) Cooking

c) Fermentation
d) Distillation
e) I don't know
9. Traditional way of assessing the alcohol content in Mezcal
a) Rubbing
b) Breathalyzer
c) Venenciar
d) Heating
e) I don't know
10. Some brands of Mezcal are characterized by containing:
a) Chilis
b) Maguey Worm
c) Pieces of wood
d) Barley
e) I don't know
11. The average alcohol content in Mezcal is:
a) 20% V/V
b) 45% V/V
c) 100% V/V
d) 38% V/V
e) I don't know
12. Usually, in which container the Mezcal is consumed?
a) Bowl
b) Pot
c) Cup
d) Caligüey
e) I don't know
13. Which of the following names corresponds to a maguey?
a) Maguey escarabajo
b) Maguey espadín
c) Maguey toro
d) Maguey marino
e) I don't know
14. Some people tend to consume Mezcal accompanied by:
a) Salt and lemon
b) Worm salt and orange
c) Chili powder
d) Oregano
e) I don't know
15. What is the name of Mezcal made from different agaves?
a) Double
b) Simple
c) Assembly
d) Mixed
e) I don't know

Please, select the option you think is correct for each question.

1. What state is the origin of Tequila?
a) Sonora
b) Jalisco
c) Tabasco
d) Durango
e) I don't know
2. What does the term “reposed” Tequila refer to?
a) That has been incubated
b) What has been stored in casks
c) What has been purified
d) I don't know
3. What agave is used to make Tequila?
a) Agave cenizo
b) Agave papalote
c) Agave manso
d) Agave azul
e) I don't know
4. What is a category of Tequila?
a) Red
b) National
c) Aged
d) Industrial
e) I don't know
5. What element protects the production of Tequila?
a) ISO Norm
b) Intellectual Protection Norm
c) Denomination of origin
d) Sanity Norm
e) I don't know
6. What does the term congeners refer to?
a) Little children
b) Volatile compounds
c) Liquids
d) Plants
e) I don't know
7. It is the scientific name of the agave that is used to make Tequila
a) *Agave angustifolia*
b) *Agave durangensis*
c) *Agave tequilana weber* var. blue
d) *Agave maximiliana*
e) I don't know
8. It is one of the differences between Mezcal and Tequila
a) The raw material
b) Cooking
c) Fermentation
d) Distillation
e) I don't know

9. Container in which tequila is traditionally served
a) Bowl
b) Cup
c) Caballito
d) Pot
e) I don't know
10. It is the process where the greatest amount of volatile compounds that give aromatic notes to Tequila are generated.
a) Cooking
b) Distillation
c) Fermentation
d) Grinding
e) I don't know
11. What does 100% agave mean?
a**) What is made of pure agave**
b) What is done in Tequila
c) What is artificial
d) What is international
e) I don't know
12. Necessary time to mature an agave to make Tequila
a) Between 13 and 15 years
b) Between 1 and 3 years
c) Between 20 and 25 years
d) Between 7 and 8 years
e) I don't know
13. Tequila is a beverage:
a) Foreign
b) Distilled
c) Concentrated
d) Moisturizer
e) I don't know
14. Some people tend to consume Tequila accompanied by:
a) Salt and lemon
b) Worm and orange salt
c) Chili powder
d) Oregano
e) I don't know
15. It is a drink made from Tequila and soda
a) Silk stockings
b) Cuba
c) Paloma
d) Mayan Heaven
e) I don't know
